# Trans‐gnetin H isolated from the seeds of *Paeonia* species induces autophagy via inhibiting mTORC1 signalling through AMPK activation

**DOI:** 10.1111/cpr.13360

**Published:** 2022-11-15

**Authors:** Chao Xia, Guoyan Wang, Lei Chen, Huijun Geng, Junhu Yao, Zhangzhen Bai, Lu Deng

**Affiliations:** ^1^ College of Animal Science and Technology, Northwest A&F University Yangling Shaanxi China; ^2^ College of Landscape Architecture and Arts, Northwest A&F University Yangling Shaanxi China

## Abstract

*Paeonia* is a well‐known species of ornamental plants, traditional Chinese medicines, and emerging oilseed crops. Apart from nutritional unsaturated fatty acids, the seeds of peonies are rich in stilbenes characterized by their wide‐ranging health‐promoting properties. Although the typical stilbene resveratrol has been widely reported for its multiple bioactivities, it remains uncertain whether the trimer of resveratrol trans‐gnetin H has properties that regulate cancer cell viability, let alone the underlying mechanism. Autophagy regulated by trans‐gnetin H was detected by western blotting, immunofluorescence, and quantitative real‐time PCR. The effects of trans‐gnetin H on apoptosis and proliferation were examined by flow cytometry, colony formation and Cell Counting Kit‐8 assays. Trans*‐*gnetin H significantly inhibits cancer cell viability through autophagy by suppressing the phosphorylation of TFEB and promoting its nuclear transport. Mechanistically, trans‐gnetin H inhibits the activation and lysosome translocation of mTORC1 by inhibiting the activation of AMPK, indicating that AMPK is a checkpoint for mTORC1 inactivation induced by trans‐gnetin H. Moreover, the binding of TSC2 to Rheb was markedly increased in response to trans‐gnetin H stimulation. Similarly, trans‐gnetin H inhibited the interaction between Raptor and RagC in an AMPK‐dependent manner. More importantly, trans‐gnetin H‐mediated autophagy highly depends on the AMPK‐mTORC1 axis. We propose a regulatory mechanism by which trans‐gnetin H inhibits the activation of the mTORC1 pathway to control cell autophagy.

## Graphical abstract

- Proposed a mechanism by which trans‐gnetin H inhibits the activation of the mTORC1 pathway to control cell autophagy.

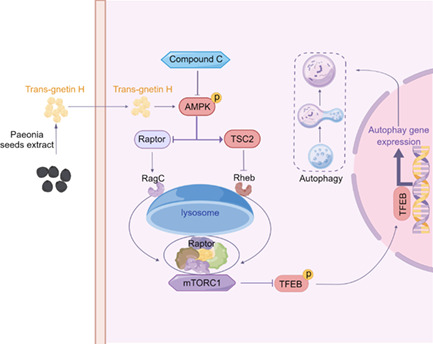

## 1 INTRODUCTION

*Paeonia* is a commonly known species of ornamental plants and traditional Chinese medicines worldwide. In the last two decades, studies have shown that the seeds of peonies are rich in unsaturated fatty acids (>90%), especially α‐linolenic acid (>40%), which has various health‐promoting properties.^1^, ^2^, ^3^ With the increasing pursuit of a healthy lifestyle, peony seed oil has been gradually accepted by consumers and authenticated as a new resource of food by the Chinese Ministry of Health. Besides nutritional unsaturated fatty acids, the bioactive constituents in peony seeds attract the attention of researchers. Recently, it has been found that the seeds of peony species are rich in stilbene compounds. Trans‐resveratrol (trans‐3,4′,5‐trihydroxystilbene), the most typical stilbene compound, is characterized by its wide‐ranging pharmacological potentials, such as antioxidant, antidiabetic, anti‐inflammatory, anti‐atherosclerotic, anticancer, estrogenic, anti‐osteoporosis, neuroprotective, cardioprotective, antiaging, and anti‐obesity properties.^4^, ^5^ To date, more than 20 stilbene compounds have been reported in the genus *Paeonia*, most of which are in glycosylated, methylated, and oligomeric forms of resveratrol.^6^, ^7^, ^8^, ^9^ Trans‐gnetin H, a trimer of resveratrol, is one of the most dominant stilbenes in peony seeds.^10^, ^11^ It is reported that resveratrol has an antitumor potential in various human tumour models.^12^, ^13^, ^14^ However, the biological function of trans‐gnetin H against cancer remains unclear. The cellular process autophagy that eliminates damaged organelles or cellular components has attracted the attention of researchers for its strong correlation with many diseases, including cancer, neurodegenerative diseases, myopathy, and heart diseases. If autophagy is found to be dysregulated, activating it can reduce symptoms and, in some cases, may even cure these disorders.^15^, ^16^ And mechanistic target of rapamycin complex 1 (mTORC1) has been well researched about autophagy.^17^ Specifically, some early autophagy‐related proteins, such as autophagy‐related protein 13 (ATG13) and unc‐51‐like kinase 1 (ULK1), can be phosphorylated and inactivated by mTORC1.^18^, ^19^, ^20^, ^21^ Moreover, mTORC1‐mediated autophagy and beclin 1 regulator 1 phosphorylation can disrupt the formation of autophagosome nucleation under nutrient‐rich conditions, subsequently the inhibition of autophagy.^22^, ^23^ Two key transcription factors EB (TFEB) and E3 (TFE3) mediate the activation of lysosome biogenesis‐related genes and restoration of autophagosome initiation.^24^ The phosphorylation of these transcription factors promote the interaction of TFEB with 14‐3‐3 protein to retain them in the cytoplasm by inhibiting nuclear translocation; the inhibition of the transcription of *TFEB* and *TFE3* genes is required for lysosomal biogenesis and autophagy.^25^, ^26^, ^27^, ^28^ Recently, some studies have suggested that the health‐promoting property of resveratrol is attributed to its ability to induce autophagy.^29^, ^30^, ^31^, ^32^ Although some evidence links resveratrol directly with autophagy, whether trans‐gnetin H regulates autophagy or not remains unclear.

The Rag and Rheb GTPases, the major Ras‐related small G proteins activating mTORC1, directly target the upstream regulators of mTORC1.^33^, ^34^ Rag GTPases should be activated for the amino acid‐dependent translocation of mTORC1. Rag‐GTPase activation is regulated by several regulators, such as SAMTOR,^35^ scavenger receptor class B member 1,^36^ GTPase‐activating protein activity toward the Rags 1/2,^37^ cellular arginine sensor for mTORC1,^38^ and SESN2.^39^ In contrast, mTORC1 responds to growth factor signalling through the phosphoinositide 3‐kinases (PI3K)‐AKT signalling pathway.^33^ The primary upstream regulator of Rheb is the tuberous sclerosis complex that suppresses mTORC1 activity by acting as a GTPase‐activating protein of Rheb.^40^, ^41^ The phosphorylation of tuberous sclerosis 2 (TSC2) by AKT, AMP‐activated protein kinase (AMPK), extracellular regulated protein kinases (ERK), or p90 ribosomal S6 kinase serves as an extracellular signal in regulating the activity of TSC2 on Rheb.^42^, ^43^ AMPK is well known as a crucial regulator of intracellular metabolic homeostasis in sensing cellular energy fluctuations and transmitting the information of glucose availability and energy levels to the mTORC1 pathway.^44^ The literature on this topic suggests that TSC2, mTOR, and regulatory‐associated protein of mTOR (Raptor) are target substrates of AMPK in regulating the mTORC1 pathway with glucose signals. TSC2 can be phosphorylated by AMPK to promote its GTPase activity and inhibit the activation of Rheb and mTORC1 signalling under energy‐depleted conditions. The mTORC1 pathway can also be directly regulated by AMPK by phosphorylating Raptor and mTOR.^45^ Current research on mTORC1 mainly focuses on the role of amino acids, growth factors, glucose, etc. in regulating the activation of mTORC1, and it remains uncertain whether other bioactive molecules, for example, trans‐gnetin H, have any role in the mTORC1 pathway.

In this study, we found that trans‐gnetin H regulates autophagy by inhibiting phosphorylation and promoting the nuclear transport of TFEB. We further discovered that trans‐gnetin H inhibited the activation and lysosome translocation of the mTORC1 pathway. Moreover, we explored the mechanisms underlying trans‐gnetin H treatment enhanced the interaction between TSC2 and Rheb and inhibited the binding of Raptor and RagC by targeting the AMPK pathway. Furthermore, we found that trans‐gnetin H failed to regulate mTORC1 activation and autophagy in AMPK knockdown or inhibition cells, thereby suggesting that the regulation of AMPK activation is an important mechanism for the activation of mTORC1 by trans‐gnetin H.

## 2 MATERIALS AND METHODS

### 2.1 Reagents and plasmids

Trans‐gnetin H (determined >99%) was isolated from the seeds of peony. In cell experiments, trans‐gnetin H was dissolved by dimethyl sulfoxide (DMSO) to a certain concentration. Glucose (D9559), Rapamycin (V900930), Torin1 (475991), and Insulin (I0310000) were obtained from Sigma–Aldrich (MO, USA). Bafilomycin A1 (S1413) and Compound C (S7840) were obtained from Selleck Chemicals (Houston, USA). PBS and trypsin were purchased from HyClone (UT, USA). RPMI 1640 medium, β‐mercaptoethanol, penicillin, foetal bovine serum (FBS), streptomycin, amino acids (AA, 50X), and DMEM (glucose‐free) were bought from Gibco (Utah, USA). The amino acid‐free DMEM was purchased from Genetimes Technology (Shanghai, China). TB Green qRT‐PCR kit (RR820A), PrimeScript™ RT reagent Kit (RR047A), and Trizol reagent were obtained from Takara (Dalian, China). Flag‐TFEB, Flag‐AMPK, Myc‐Raptor, HA‐TSC2, Flag‐Rheb and Flag‐RagC were provided by P. Wang (Tongji University, Shanghai, China).

### 2.2 Cell culture

Human non‐small cell lung cancer cells (H1299 and A549), human colorectal carcinoma cells (HCT116 and HT29), human cervical cancer cells (HeLa), human hepatocarcinoma cells (HepG2) and human breast carcinoma cells (MDA‐MB‐231) were purchased from National Science & Technology Infrastructure (NSTI, Shanghai, China), and H1299, HT29, A549, HeLa were cultured in RPMI 1640 medium with 10% FBS following the ATCC guidelines, HCT116, HepG2, and MDA‐MB‐231 were cultured in DMEM medium with 10% FBS following the ATCC guidelines.

### 2.3 Quantitative real‐time PCR (qRT‐PCR) analysis

The isolation of total RNA was performed using TRIzol reagent and treated with RNase‐free DNase. Then, we conducted reverse transcription and determined which RNA purity and concentration through the NanoDrop 2000C Spectrophotometer (Thermo Fisher Scientific, USA). qRT‐PCR analysis was conducted in technical triplicate in a Roche LightCycler®96 qRT‐PCR system (Roche, Germany). The internal control gene was. The comparative Ct method was used to determine the relative levels of target mRNA. The procedures of qRT‐PCR experiments as follows: 30 s at 95°C, followed by 40 cycles of 5 s at 95°C and 30 s at 60°C. For qRT‐PCR assay, primers of target genes were used as listed in Table 1.

**TABLE 1:** Primer sequences for qRT‐PCR

Gene	Forward primer sequence (5′ → 3′)	Reverse primer sequence (5′ → 3′)
*CTSA*	CAGGCTTTGGTCTTCTCTCCA	TCACGCATTCCAGGTCTTTG
*CTSB*	AGTGGAGAATGGCACACCCTA	AAGAAGCCATTGTCACCCCA
*CTSD*	AACTGCTGGACATCGCTTGCT	CATTCTTCACGTAGGTGCTGGA
*GBA*	TGGGTACCCGGATGATGTTA	AGATGCTGCTGCTCTCAACA
*GLA*	AGCCAGATTCCTGCATCAGTG	ATAACCTGCATCCTTCCAGCC
*LAMP1*	ACGTTACAGCGTCCAGCTCAT	TCTTTGGAGCTCGCATTGG
*MCOLN1*	TTGCTCTCTGCCAGCGGTACTA	GCAGTCAGTAACCACCATCGGA
*NEU1*	CAGCACATCCAGAGTTCCGAGT	TGTCTCTTTCCGCCATGAGGT
*NAGLU*	CAGAAGGAAGGAGCAGGAGT	ATGTTCCCGAGGCTGTCAC
*SCPEP1*	GATCTCCCCTGTTGATTCGGT	AGCCCCTTATTTACGGCATTC
*GALNS*	TTGTCGGCAAGTGGCATCT	CCAAACCACTCATCAAATCCG
*ATP6V1H*	GGAAGTGTCAGATGATCCCCA	CCGTTTGCCTCGTGGATAAT
*TMEM55B*	GTTCGATGCCCCTGTAACTGTC	CCCAGGTTGATGATTCTTTTGC
*PSAP*	GCCAACAGTGAAATCCCTTCC	TCAGTGGCATTGTCCTTCAGC
*GAPDH*	CAACGAATTTGGCTACAGCA	AGGGGTCTACATGGCAACTG

### 2.4 Autophagy analysis

Autophagy analysis was conducted as the previous description.^46^ Briefly, GFP‐LC3 plasmid was used to transfect H1299 cells and then treated with trans‐gnetin H and resveratrol for 6 h. Paraformaldehyde (4%) was used to fix the cells and maintained for 15 min. The permeabilized of fixed cells was performed using 0.5% Triton X‐100 in PBS for another 15 min. Sequentially, it was blocked at room temperature using 1% BSA for 1 h and stained with DAPI. Finally, the GFP‐LC3‐containing puncta were measured using laser a scanning confocal microscopy (Leica, Germany).

### 2.5 Cell apoptosis

Trans‐gnetin H and resveratrol were given to H1299 cells for a 2‐h incubation. Then the cells were stained using YF®488‐Annexin V/PI apoptosis Detection Kit, after which the flow cytometry (BD FACSAria III, USA) was employed to measure apoptotic cells.

### 2.6 Cell viability assay

Ten thousand cells were seeded in each well on complete RPMI 1640 medium containing different concentrations of trans‐gnetin H and resveratrol for the indicated time, then 100 ml of fresh medium containing 10% CCK8 reagent was given to replace the original medium for a 3‐h incubation at the temperature of 37°C. Finally, the Synergy HT microplate reader (Bio‐Tek, USA) was used to determine the absorbance of each well at 450 nm.

### 2.7 Colony formation assay

Trans‐gnetin H and resveratrol were administered in a range of concentrations to the well of 6‐well plates containing 1000 to 2000 H1299 cells for a 6‐h incubation, followed by a 7‐d incubation in RPMI‐1640 medium with 10% FBS. Sequentially, paraformaldehyde (4%) was used to fixed cells. After a wash with PBS, the cells were finally stained with crystal violet.

### 2.8 Western‐blotting (WB)

The WB protocol was the same as our previous work.^47^ The primary antibodies Actin (20536‐1‐AP), LC3II (18725‐1‐AP), p62 (18420‐1‐AP) were purchased from Proteintech; TFEB (37785 S), pT389‐S6K (9234 S), p‐S6 (4858 S), S6K (9202 S), S6 (2217 S), p‐AMPKα (50081 S), AMPKα (5832 S), Raptor (2280 S) and TSC2 (D93F12) were purchased from Cell Signaling Technology; Flag (db7002) and HA (db2603) were purchased from Hangzhou Bio Technolog. The secondary antibodies were obtained from Sigma. Finally, the imaging System (Bio‐Rad, Hercules, CA, USA) and ImageJ software were applied to captured and quantify proteins, respectively.

### 2.9 Co‐immunoprecipitation (Co‐IP)

Co‐IP was conducted according to the previous protocol.^46^ In brief, The CHAPS lysis buffer was added to lyse the transfected H1299 cells under the condition of sonication for 10 min. Then a 15‐min microcentrifuge was used to isolate the soluble fraction at 12,000 rpm at 4°C. After centrifugation, the cell debris was separated from cell lysates and incubated in anti‐Flag M2 beads for 2–3 h. Then a thorough washing was conducted. The beads were resolved on SDS‐PAGE gel electrophoreses after being boiled and sequentially subjected to immunoblotting analysis. Finally, the Bio‐Rad imaging system (Hercules, CA, USA) was employed to detected proteins.

### 2.10 siRNA knockdown

siRNA and siRNAs for AMPKα served as non‐specific controls. Lipofectamine 2000 regent (Invitrogen, CA, USA) and siRNA oligonucleotides were used to transfect H1299 cells. The siRNAs were used as follows:

- si AMPKα: 5′‐GTTGCCTACCATCTCATAATA‐3′;
- si TSC2: 5′‐CGACGAGTCAAACAAGCCAAT‐3′.

### 2.11 Immunofluorescence

H1299 cells were seeded and incubated for 24 h on fibronectin‐coated glass coverslips in 24‐well plates, after which the cells were transfected in complete RPMI 1640 medium or starved in amino acid‐free DMEM medium containing trans‐gnetin H before stimulation with amino acids. Then, we rinsed the cells once with PBS before fixing them in paraformaldehyde solution (4%) for 15 min. The fixed cells were rinsed with PBS two times after the permeabilization with 0.1% Triton X‐100. After a 1‐h block with blocking buffer (0.3% BSA in PBS), the coverslips were incubated in a primary antibody at 4°C overnight. Next, we rinsed the coverslips two times with blocking buffer and incubated in secondary antibodies at room temperature in the dark for 1 h before the amplification of tyramide signal. The glass coverslips were mounted using Mowiol and examined using a Zeiss LSM 510 Meta confocal system.

### 2.12 Statistical analysis

Data analysis was performed on GraphPad Prism 9.0 software and the result was expressed as mean ± SEM. Statistical analysis, including unpaired one‐tailed or two‐tailed Student's *t*‐test and one‐way or two‐way analysis of variance, were conducted with *p* value at 0.05 level to determine the statistically significance. *p* values less than 0.05, 0.01, and 0.001 were marked with *, **, and *** in the graphed data, respectively.

## 3 RESULTS

### 3.1 Trans‐gnetin H regulates cellular autophagy via TFEB

**FIGURE 1:**
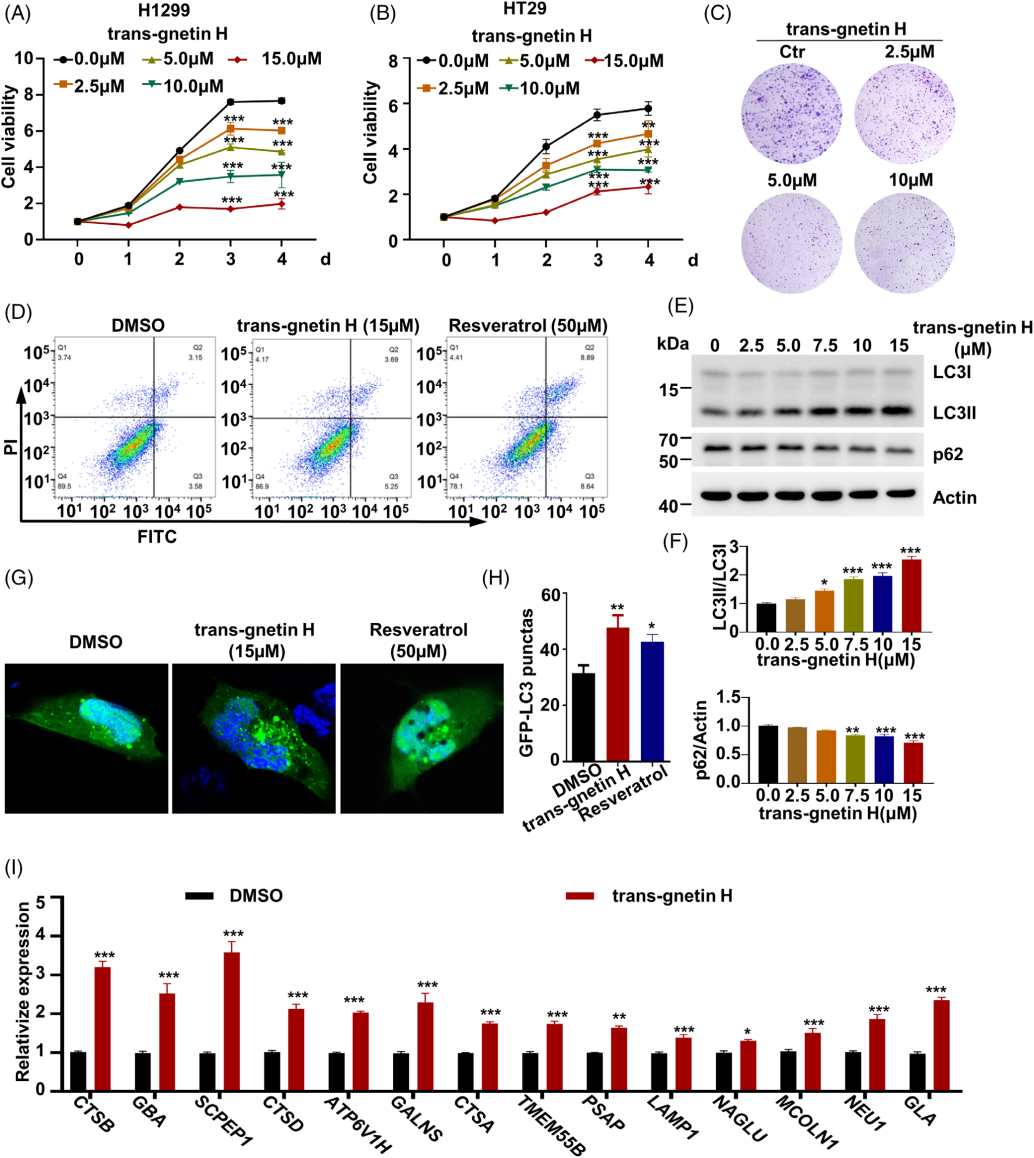
Trans‐gnetin H regulates cellular autophagy via TFEB. (A, B) H1299 (A) and HT29 (B) cells were treated with different concentrations of trans‐gnetin H for indicated time, the viability of cells was detected by CCK8, all samples were normalized to cell number and conducted with three independent replicates. (C) H1299 cells were treated with different concentrations of trans‐gnetin H, the viability of cells was detected by colony formation. (D) H1299 cells were treated with trans‐gnetin H (15 μM) and resveratrol (50 μM) for 2 h, and cell apoptosis was examined using flow cytometer. (E, F) H1299 cells were treated with different concentrations of trans‐gnetin H for 6 h after which autophagy was detected by WB (E), and quantified by ImageJ, *n* = 3 (F). (G, H) H1299 cells were treated with trans‐gnetin H (15 μM) and resveratrol (50 μM) for 6 h, after which cell autophagy was analysed by examining GFP‐LC3 puncta (G) and quantified (H). (I) H1299 cells were treated with trans‐gnetin H (10 μM) for 6 h, and the expression of *CTSB*, *GBA*, *SCPEP1*, *CTSD*, *ATP6V1H*, *GALNS*, *CTSA*, *TMEM55B*, *PSAP*, *LAMP1*, *NAGLU*, *MCOLN1*, *NEU1*, and *GLA* was detected by qRT‐PCR, *n* = 3.

Firstly, the purity of trans‐gnetin H is determined >99% with HPLC result (Figure S1). To determine whether trans‐gnetin H has any effect on cellular functions, we treated a variety of cancer cell lines including H1299, A549, HT29, HCT116, HepG2, HeLa, and MDA‐MB‐231 with trans‐gnetin H and we found that Trans‐gnetin H showed the more significant effect in H1299 and HT29 cells (Figures 1A,B and S2A). The viability was reduced by more than 50% when the trans‐gnetin H concentration reached 15 μM (Figure 1A,B). As a positive control, we found that resveratrol suppressed the cell proliferation (Figure S2B–E), and our findings suggest that trans‐gnetin H is more potent than resveratrol in inhibiting tumour cell proliferation (Figure S2D,E). According to the colony formation assay, we found that trans‐gnetin H suppressed the colony formation of H1299 cells in a dose‐dependent manner (Figure 1C). Consistently, we found that resveratrol was also able to inhibit colony formation of cell, but its effect was significantly less than that of trans‐gnetin H (Figure S2F,G). These results suggest that trans‐gnetin H is able to inhibit the proliferation of a variety of tumour cells.

Autophagy and apoptosis are widely known as major mechanisms involved in cell viability. Therefore, we conducted apoptosis and autophagy assays of H1299 cells treated with trans‐gnetin H. We did not observe a significant apoptosis effect on H1299 cells when they were treated with 15 μM trans‐gnetin H, while as a positive control, resveratrol promoted the cell apoptosis (Figure 1D). Moreover, a sharp increasing level of autophagy marker LC3II^48^ was observed with the treatment of differential concentrations of trans‐gnetin H (Figure 1E,F), which tentatively indicated that trans‐gnetin H mainly affects cell viability through the autophagy mechanism. Nevertheless, the application of trans‐gnetin H (15 μM) and resveratrol (50 μM) led to the promotion of autophagy, as an increasing number of punctate GFP‐LC3 foci were observed after bafilomycin A1 treatment (Figure 1G,H).

TFEB is the key factor mediating the activation of lysosome biogenesis‐related genes and the restoration of autophagosome initiation.^24^ Thus, the function of trans‐gnetin H in regulating lysosome biogenesis was examined. We found that the expressions of *CTSB*, *GBA*, *SCPEP1*, *CTSD*, *ATP6V1H*, *GALNS*, *CTSA*, *TMEM55B*, *PSAP*, *LAMP1*, *NAGLU*, *MCOLN1*, *NEU1*, and *GLA* were significantly enhanced after a 6‐h trans‐gnetin H treatment (Figure 1I), indicating that trans‐gnetin H promotes autophagy without inducing apoptosis.

### 3.2 Trans‐gnetin H inhibits mTORC1 activation

**FIGURE 2:**
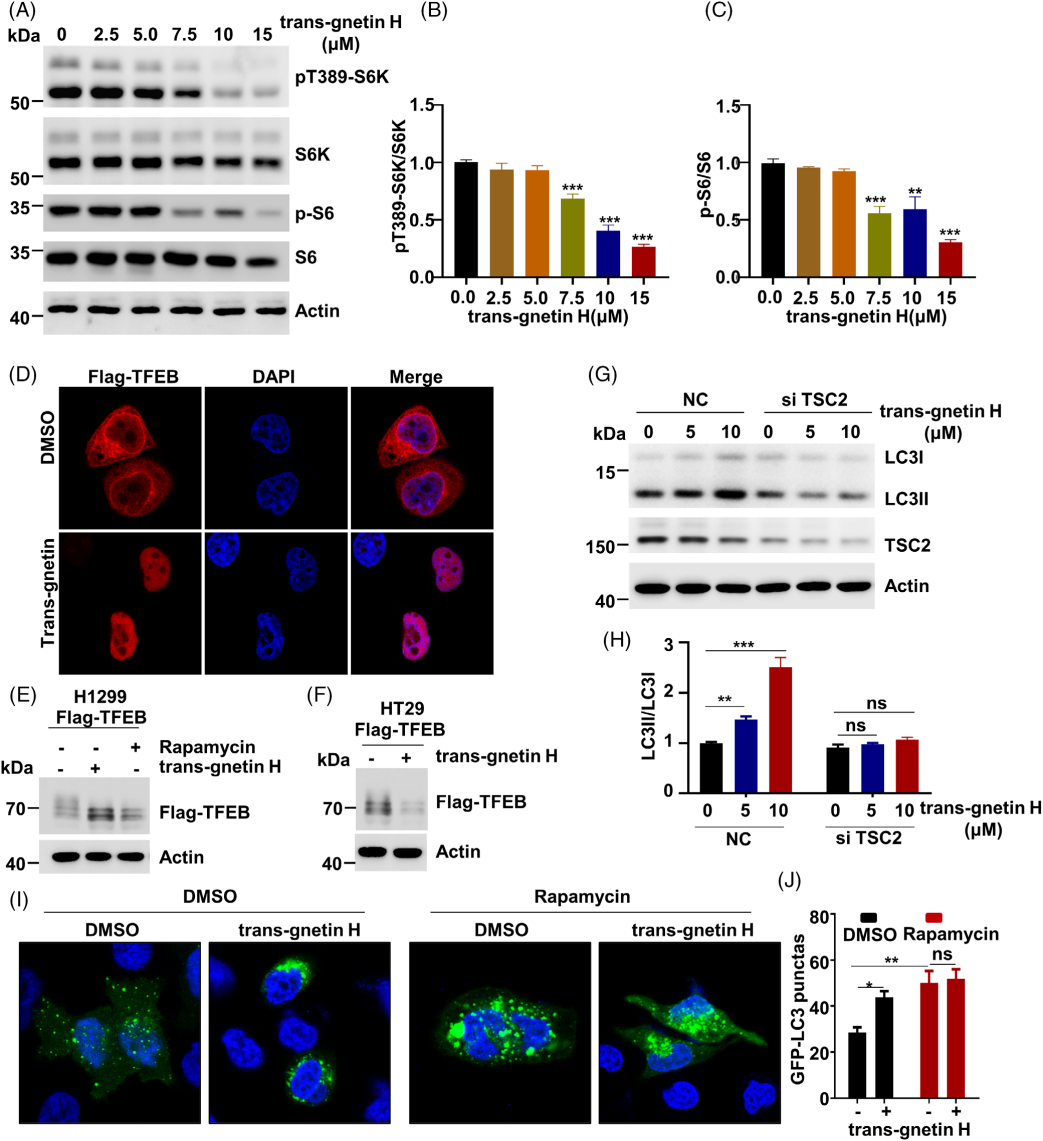
Trans‐gnetin H inhibits mTORC1 activation. (A–C) H1299 cells were treated with different concentrations of trans‐gnetin H for 2 h, the indicated proteins were detected by WB (A), and quantified by ImageJ, *n* = 3 (B, C). (D) H1299 cells were transfected with Flag‐TFEB and treated with trans‐gnetin H for 6 h, and nuclear localization of TFEB was observed by immunofluorescence. (E, F) H1299 (E) and HT29 (F) cells were treated with trans‐gnetin H (10 μM) for 6 h, the phosphorylation of TFEB was detected by WB. (G, H) TSC2 was knockdown in H1299 cells and cells were treated with different concentrations of trans‐gnetin H for 6 h, after which cell autophagy was detected by WB (G), and quantified by ImageJ, *n* = 3 (H). (I, J) H1299 cells were treated with trans‐gnetin H (10 μM) or in combination with rapamycin (10 nM) for 6 h, after which cell autophagy was analysed by examining GFP‐LC3 puncta (I) and quantified (J).

mTORC1 is a negative regulator of autophagy, which transcriptionally regulates genes related to lysosome biogenesis by directly phosphorylating TFEB.^24^ Thus, we examined whether trans‐gnetin H induced autophagy by inhibiting mTORC1 activation. To investigate whether trans‐gnetin H had a regulatory effect on mTORC1 activation, H1299 cells were treated with trans‐gnetin H for 2 h. The mTORC1 activity was monitored by measuring the phosphorylation of S6K1 at Thr389 (pT389‐S6K) and the phosphorylation of S6, which are well‐characterized mTORC1‐dependent phosphorylation sites. As expected, we found that the activation of mTORC1 was inhibited by the phosphorylation of S6K1 and S6 in a dose‐dependent manner when treated with trans‐gnetin H (Figure 2A–C). Given that trans‐gnetin H induces autophagy, we determined whether trans‐gnetin H plays any role in the phosphorylation of TFEB. We found that the nuclear transport of TFEB, a key marker of autophagy,^21^, ^26^, ^49^, ^50^ was almost completely enhanced with trans‐gnetin H treatment (Figure 2D). Consistent with these findings, our results confirmed that trans‐gnetin H stimulation led to a remarkable suppression of the phosphorylation of TFEB in H1299 and HT29 cells (Figures 2E,F and S3A,B). To further verification whether mTORC1 is involved in the trans‐gnetin H‐mediated regulation of cell autophagy, a remarkable finding was that the effect of trans‐gnetin H on autophagy was completely abrogated in TSC2 knockdown cells (Figure 2G,H).

Further, to examine whether the effect of trans‐gnetin H on autophagy occurs in an mTORC1‐dependent manner, we treated the cells with rapamycin, an inhibitor of mTORC1. The results showed that trans‐gnetin H treatment did not inhibit rapamycin‐mediated autophagy as detected by the appearance of GFP‐LC3II puncta (Figure 2I,J). Based on all these findings, we concluded that mTORC1‐mediated cell autophagy, were negatively regulated by trans‐gnetin H.

### 3.3 Trans‐gnetin H inhibits mTORC1 activation in an insulin/amino acid/glucose‐sensitive manner

**FIGURE 3:**
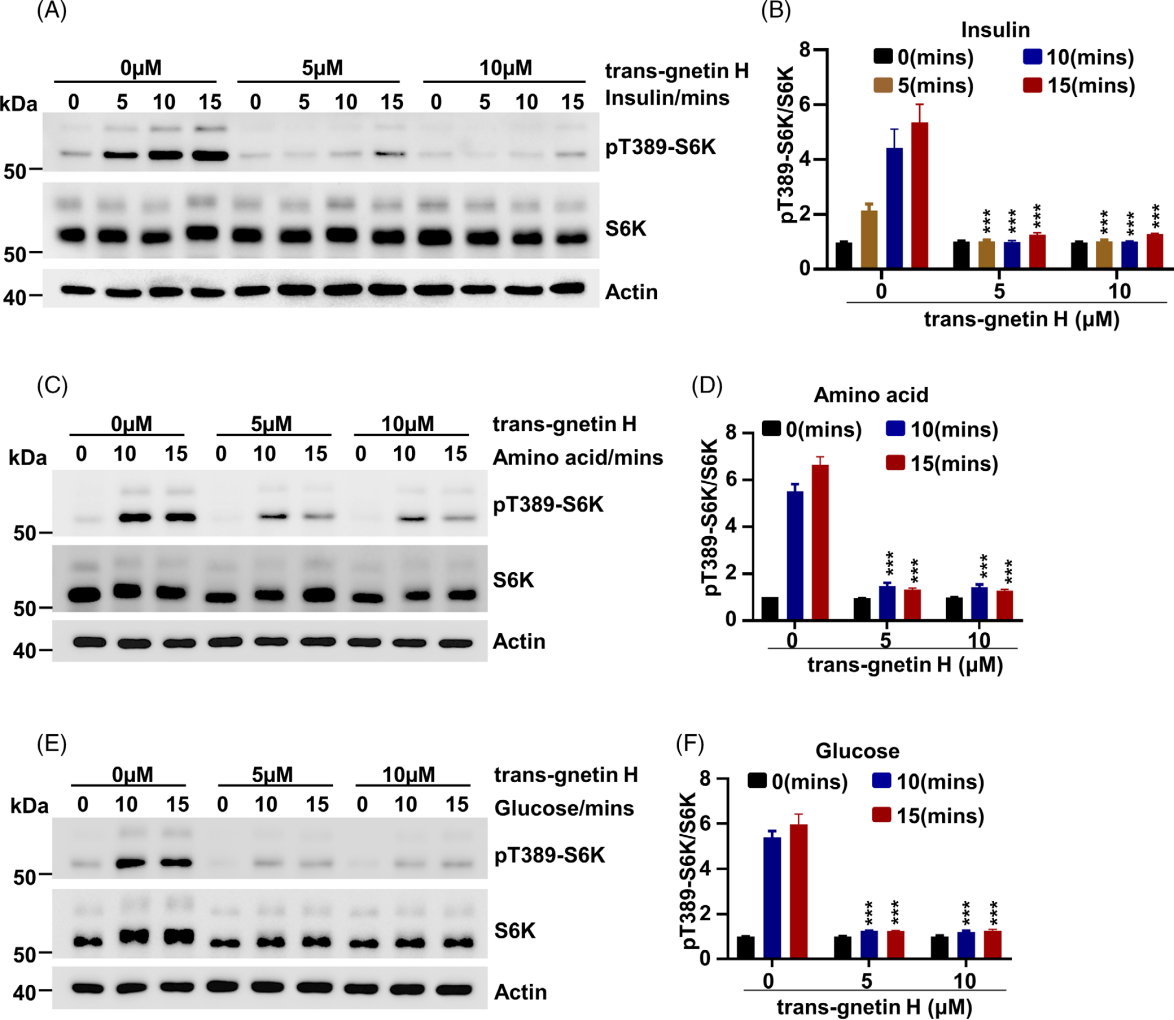
Trans‐gnetin H inhibits mTORC1 activation in an insulin/amino acid/glucose‐sensitive manner. (A, B) H1299 cells were incubated in serum‐free DMEM with different concentrations of trans‐gnetin H for 2 h and then stimulated with Insulin (100 nM) for the indicated time, the indicated proteins were detected by WB (A), and quantified by ImageJ, *n* = 3 (B). (C, D) H1299 cells were incubated in amino acid‐free DMEM with different concentrations of trans‐gnetin H for 2 h and then stimulated with amino acids (AA, 50×) for the indicated time, the indicated proteins were detected by WB (C), and quantified by ImageJ, *n* = 3 (D). (E, F) H1299 cells were incubated in glucose‐free DMEM with different concentrations of trans‐gnetin H for 2 h and then stimulated with glucose for the indicated time, the indicated proteins were detected by WB (E), and quantified by ImageJ, *n* = 3 (F).

Activation of mTOR is closely associated with environmental stimuli such as insulin‐, amino acid‐, and glucose‐related signals.^51^ Given that trans‐gnetin H significantly inhibited the activation of mTORC1 (Figure 2), its role in insulin‐, amino acid‐, or glucose‐induced mTORC1 activation was further investigated. Our data showed that insulin, amino acid, and glucose stimulation resulted in a significant increase in pT389‐S6K (Figure 3A–F). Importantly, we found that trans‐gnetin H treatment blocked the insulin‐, amino acid‐, and glucose‐mediated mTORC1 activation (Figure 3A–F). These results collectively demonstrated that the function of trans‐gnetin H is to inhibit mTORC1 activation in an insulin‐, amino acid‐, or glucose‐sensitive manner.

### 3.4 Trans‐gnetin H inhibits mTORC1 activation by inducing the activation of AMPK

**FIGURE 4:**
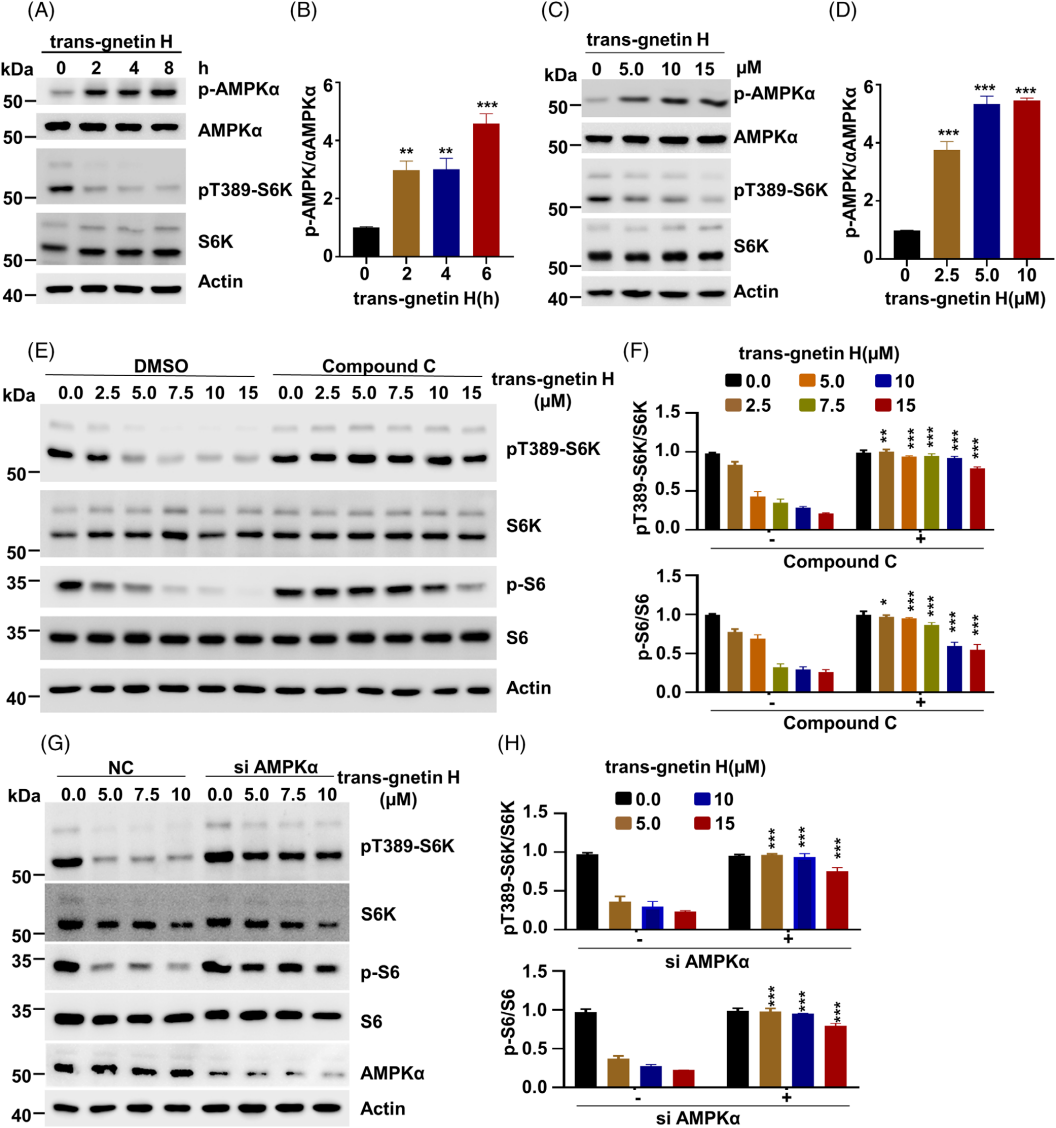
Trans‐gnetin H inhibits the mTORC1 activation by inducing the activation of AMPK. (A, B) H1299 cells were treated with trans‐gnetin H (10 μM) for indicated time, the indicated proteins were detected by WB (A), and quantified by ImageJ, *n* = 3 (B). (C, D) H1299 cells were treated with different concentrations of trans‐gnetin H for 2 h, the indicated proteins were detected by WB (C), and quantified by ImageJ, *n* = 3 (D). (E, F) H1299 cells were treated with different concentrations of trans‐gnetin H or in combination with Compound C for 2 h, after which the indicated proteins were detected by WB (E), and quantified by ImageJ, *n* = 3 (F). (G, H) AMPKα was knockdown in H1299 cells, and cells were treated with different concentrations of trans‐gnetin H for 6 h, the indicated proteins were analysed (G), and quantified by ImageJ, *n* = 3 (H).

We further elucidated the molecular mechanisms underlying trans‐gnetin H‐induced mTORC1 inactivation. It is reported that many health‐promoting properties of resveratrol on metabolic function depend on AMPK.^52^, ^53^, ^54^ We next sought to determine whether trans‐gnetin H is involved in AMPK activation. To this end, H1299 cells were treated with trans‐gnetin H. We found that the activation of AMPK was significantly induced in response to trans‐gnetin H treatment in a time‐dependent manner (Figure 4A,B). Consistently, the activation of AMPK was also controlled by trans‐gnetin H in a dose‐dependent manner (Figure 4C,D). Next, we examined the role of AMPK in regulating trans‐gnetin H‐dependent mTORC1 inactivation and found that the treatment with the AMPK inhibitor Compound C remarkably reversed the suppressive function of trans‐gnetin H on mTORC1 activation (Figure 4E,F). Moreover, the knockdown of AMPK with specific siRNA showed that the depletion of AMPK markedly blocked trans‐gnetin H‐mediated mTORC1 inactivation, indicating that AMPK is required for this process (Figure 4G,H).

### 3.5 Trans‐gnetin H regulates the interaction between AMPK and Raptor/TSC2

**FIGURE 5:**
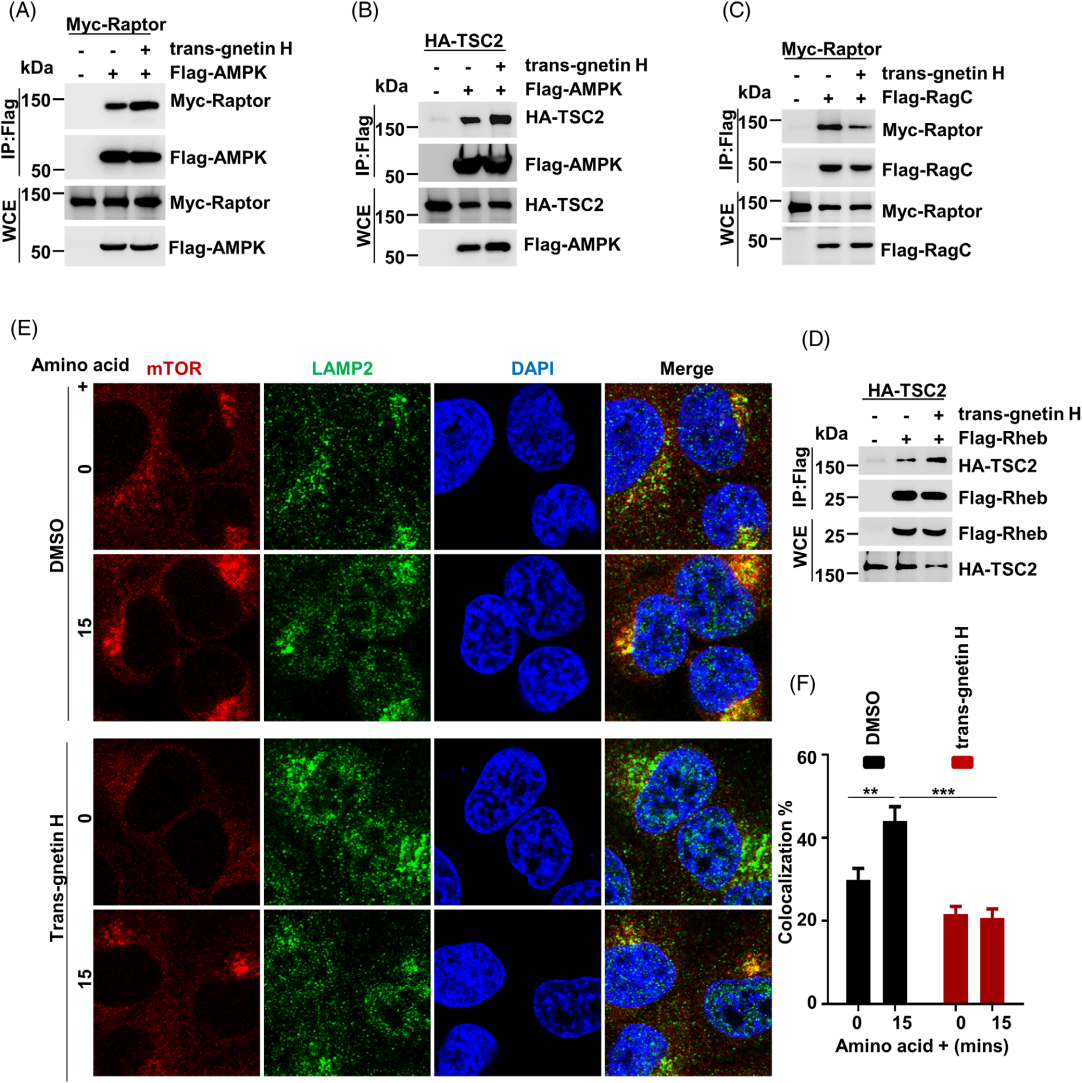
Trans‐gnetin H regulates the interaction between AMPK and Raptor/TSC2. (A) Flag‐AMPK and Myc‐Raptor were overexpressed in H1299 cells, and cells were treated with trans‐gnetin H (10 μM) for 6 h, the interaction between Flag‐AMPK and Myc‐Raptor was detected by Co‐IP assay. (B) Flag‐AMPK and HA‐TSC2 were overexpressed in H1299 cells, and cells were treated with trans‐gnetin H (10 μM) for 6 h, the interaction between Flag‐AMPK and HA‐TSC2 was detected by Co‐IP assay. (C) Flag‐RagC and Myc‐Raptor were overexpressed in H1299 cells, and cells were treated with trans‐gnetin H (10 μM) for 6 h, the interaction between Flag‐RagC and Myc‐Raptor was detected by Co‐IP assay. (D) Flag‐Rheb and HA‐TSC2 were overexpressed in H1299 cells, and cells were treated with trans‐gnetin H (10 μM) for 6 h, the interaction between Flag‐Rheb and HA‐TSC2 was detected by Co‐IP assay. (E, F) H1299 cells were treated with trans‐gnetin H (10 μM) for 6 h. The co‐localization of mTOR and LAMP2 were detected by immunofluorescence, and the quantification was carried out on at least 10 cells per condition from three independent experiments.

A previous study revealed that TSC2 and Raptor are two key substrates of AMPK regulating the mTORC1 pathway.^55^ To clarify the mechanism by which trans‐gnetin H regulates mTORC1 activation, we investigated the relation between TSC2/Raptor and AMPK using the Co‐IP assay. We found that ectopically expressed AMPK interacted with TSC2/Raptor; the interaction between TSC2/Raptor and AMPK was markedly enhanced by trans‐gnetin H treatment (Figure 5A,B). Next, we found that trans‐gnetin H disrupts the interaction between Raptor and RagC (Figure 5C). In line with the previous results, our data further showed that trans‐gnetin H treatment promoted the binding of Rheb and TSC2 (Figure 5D). These results demonstrated that AMPK plays a key role in trans‐gnetin H‐mediated mTORC1 activation by regulating the interaction between Rheb (RagC) and TSC2 (Raptor).

Existing evidence indicates that mTOR translocation from the cytosol to the lysosome is necessary for the amino acid‐induced activation of mTORC1^56^, ^57^ and that mTORC1 translocation relies on the interaction between Raptor and RagC.^58^ Therefore, we investigated the function of trans‐gnetin H on the lysosomal localization of mTOR induced by amino acids. As previously reported, our findings also showed that mTOR was localized to lysosomes after the stimulation of amino acids. Moreover, trans‐gnetin H treatment significantly inhibited the co‐localization of mTOR with the lysosomal marker LAMP2 (Figure 5E,F).

### 3.6 Trans‐gnetin H induces autophagy by inhibiting the AMPK pathway

**FIGURE 6:**
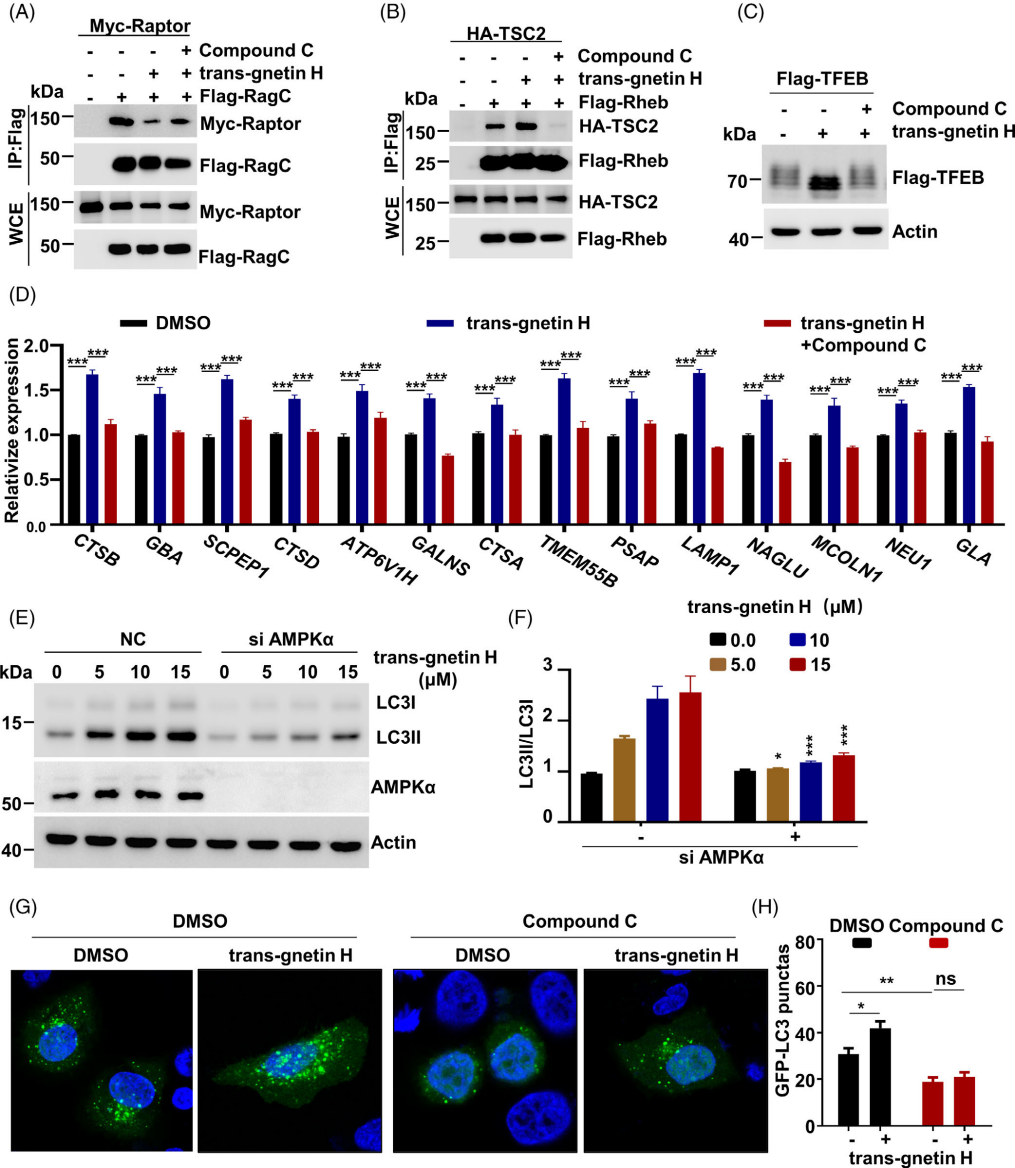
Trans‐gnetin H induces autophagy by inhibiting AMPK pathway. (A) Flag‐RagC and Myc‐Raptor were overexpressed in H1299 cells, and cells were treated with trans‐gnetin H (10 μM) or Compound C for 6 h, the interaction between Flag‐RagC and Myc‐Raptor was detected by Co‐IP assay. (B) Flag‐Rheb and HA‐TSC2 were overexpressed in H1299 cells, and cells were treated with trans‐gnetin H (10 μM) or Compound C for 6 h, the interaction between Flag‐Rheb and HA‐TSC2 was detected by Co‐IP assay. (C) H1299 cells were treated with trans‐gnetin H (10 μM) or Compound C for 6 h, the phosphorylation of TFEB was detected by WB. (D) H1299 cells were treated with trans‐gnetin H (10 μM) or Compound C for 6 h, and the expression of *CTSB*, *GBA*, *SCPEP1*, *CTSD*, *ATP6V1H*, *GALNS*, *CTSA*, *TMEM55B*, *PSAP*, *LAMP1*, *NAGLU*, *MCOLN1*, *NEU1*, and *GLA* was detected by qRT‐PCR, *n* = 3. (E, F) AMPKα was knockdown in H1299 cells and cells were treated with different concentrations of trans‐gnetin H for 6 h, after which cell autophagy was detected by WB (E), and quantified by ImageJ, *n* = 3 (F). (G, H) H1299 cells were treated with trans‐gnetin H (10 μM) or Compound C for 6 h, after which autophagy was analysed by examining GFP‐LC3 puncta (G) and quantified (H).

To understand how AMPK regulates trans‐gnetin H‐induced autophagy, we next examined whether the interaction between Rheb (RagC) and TSC2 (Raptor) mediated by trans‐gnetin H was regulated by AMPK. Interestingly, the treatment of Compound C resulted in a considerable reduction in the binding of Rheb to TSC2 and an increase in the binding of RagC and Raptor (Figure 6A,B). We found that Compound C inhibited trans‐gnetin H‐induced phosphorylation of TFEB (Figure 6C), indicating that trans‐gnetin H decreases the activation of mTORC1 and TFEB in an AMPK‐sensitive manner. Moreover, we also identified the effects of AMPK in trans‐gnetin H‐induced autophagy. In comparison with the WT cells, the inhibition of the AMPK pathway by Compound C significantly suppressed the expression of lysosomal biogenesis‐related genes and abolished the effects on *CTSB*, *GBA*, *SCPEP1*, *CTSD*, *ATP6V1H*, *GALNS*, *CTSA*, *TMEM55B*, *PSAP*, *LAMP1*, *NAGLU*, *MCOLN1*, *NEU1*, and *GLA* expressions induced by trans‐gnetin H (Figure 6D). We examined whether AMPK is involved in the trans‐gnetin H‐mediated regulation of cell autophagy. A remarkable finding was that the effect of trans‐gnetin H on autophagy was completely abrogated in AMPK knockdown cells (Figure 6E,F). Consistently, our data demonstrated that AMPK inhibition led to a reduction of GFP‐LC3 punctate foci, and the trans‐gnetin H‐induced autophagy was almost fully restored in AMPK inhibition cells; however, this effect was not observed in WT cells (Figure 6G,H). Based on these findings, we speculated that AMPK is involved in trans‐gnetin H‐dependent mTORC1 inactivation.

## 4 DISCUSSION

In this study, we revealed that mTORC1 is targeted by trans‐gnetin H, which is proved by the fact that mTORC1 activity is effectively inhibited by trans‐gnetin H in various human cancer cell lines. This study also gives a molecular explanation for the positive effects of trans‐gnetin H, which are mTORC1‐dependent autophagy activation and decreased viability of cancer cells. Our findings showed the potential use of trans‐gnetin H in mTORC1‐related diseases, including neurodegenerative diseases, cancer, and diabetes.

Resveratrol is widely regarded as one of the promising natural anticancer agents because of its remarkable inhibitory activity against cancer cells.^59^ Considerable attention focuses on the development or identification of newly derived resveratrol to improve their bio‐efficacy and bioavailability.^60^ Trans‐gnetin H is one of the most dominant stilbenes in peony seeds.^10^, ^11^ Therefore, we investigated the bioactivity of peony‐isolated trans‐gnetin H in this study, discovered the antitumor activity, and determined the underlying mechanism of trans‐gnetin H against cancer. However, the bioactivity of trans‐gnetin H, but not cis‐gnetin, was investigated in our study. Previous research reports suggest that trans‐gnetin H is less stable than cis‐gnetin H.^60^ It is not clear whether its effect is better than that of trans‐gnetin H, which requires further study.

Our findings suggest that trans‐gnetin H is more potent than resveratrol in inhibiting tumour cells proliferation. Consistent with previous studies, trans‐gnetin H possesses cytotoxic properties against various human cancer cell lines.^61^, ^62^, ^63^ In our study, we found that trans‐gnetin H was able to inhibit the cell viability of a variety of cancer cell lines, especially H1299 and HT29. Generally, the ability to trigger apoptosis is believed to be a crucial reason for the effectiveness of an anticancer agent. However, we did not observe the function of trans‐gnetin H in inducing apoptosis of cancer cells, which can be explained by the changes in experimental conditions. The concentration of trans‐gnetin H used in this study was 15 μM; however, a previous study showed that trans‐gnetin H was able to cause apoptosis in BT20 and A549 cells at a dose of 100 μM.^60^ The different types of cell lines used may also explain these contradictory observations.

TFEB is a major transcriptional regulator of lysosomal biogenesis and autophagy genes. It has been shown that TFEB directly binds to the coordinated lysosomal expression and regulation (CLEAR) motif elements in the promoter regions of many autophagy genes, thereby inducing autophagosome biogenesis and autophagosome‐lysosome fusion.^50^, ^64^, ^65^ mTORC1‐mediated phosphorylation is the primary mechanism regulating the subcellular localization of TFEB.^66^ Our results also suggest that trans‐gnetin H promoted TFEB dephosphorization and nuclear localization. Moreover, our study showed that the mTORC1 inhibitor rapamycin promoted the accumulation of GFP‐LC3 punctate foci and trans‐gnetin H treatment did not further increase GFP‐LC3 punctate foci. Also, our results showed that TSC2 knockdown restored the increase in LC3II expression caused by trans‐gnetin H. These results suggested that trans‐gnetin H promoted autophagy via inhibiting the activation of mTORC1. In addition, AMPK can trigger autophagy through direct activation of ULK1.^67^ Consistently, our study showed that knockdown of AMPKα restored the increase in LC3II expression caused by trans‐gnetin H, while AMPK inhibitor compound C treatment reduced the accumulation of GFP‐LC3 punctate foci caused by trans‐gnetin H. This suggested that trans‐gnetin H promoted autophagy via AMPKα. Altogether, these findings imply that trans‐gnetin H is essential for controlling mTORC1 activity and autophagy, which has therapeutic potential in the treatment of lung and colon cancer.

The mTORC1 regulation network has drawn great attention due to its major role in treating various serious disorders.^55^ Many studies on mTORC1 have focused on the regulation of amino acids, growth factors, or glucose‐induced mTORC1 activation.^55^ However, whether mTORC1 is activated by other bioactive molecules or not is unclear. A few studies have reported the resveratrol stimulation and regulation of mTORC1 activation.^68^, ^69^, ^70^, ^71^ Surprisingly, we found that trans‐gnetin H can effectively regulate the activation of mTORC1. Our data showed that trans‐gnetin H treatment can promote amino acid‐, insulin‐, and glucose‐induced increase in the phosphorylation levels of S6K1. Many environmental cues, such as nutrients and growth factors, can influence the regulation of organismal growth by mTORC1.^72^ However, we examined only the effect of trans‐gnetin H on amino acid‐, insulin‐, and glucose‐mediated activation of mTORC1. Whether trans‐gnetin H is involved in other signals, including lipid and stress, mediating the activation of the mTORC1 pathway requires further investigation.

Resveratrol has been reported to have the ability to activate AMPK, which can enhance the health‐promoting properties of resveratrol.^54^, ^73^, ^74^, ^75^ It seems unlikely that resveratrol directly targets AMPK even though resveratrol stimulates AMPK.^76^, ^77^ Evidence suggests that resveratrol directly targets phosphodiesterases.^78^ In our study, we observed that trans‐gnetin H, a trimer of resveratrol, activated the AMPK pathway. Since the known upstream regulators of mTORC1 are AMPKs, we detected the interaction between Rheb (RagC) and TSC2 (Raptor), and our results showed that the inhibition of mTORC1 by trans‐gnetin H was mediated by regulating the binding of Rheb (RagC) and TSC2 (Raptor). However, we did not know whether phosphodiesterases have any effect on the trans‐gnetin H‐mediated inactivation of mTORC1, and this requires further research. Moreover, we did not provide evidence that AMPK is the direct target of trans‐gnetin H, as we did not perform a structure analysis and in‐vitro binding experiments of trans‐gnetin H and AMPK, which needs to be resolved in the future.

The regulation of organismal growth mediated by mTORC1 was influenced by various environmental cues, such as nutrients and growth factors.^72^ Cell‐based investigations demonstrated that mTORC1 can be influenced by trans‐gnetin H through AMPK. However, their significance in mammalian physiology is unclear. Considering that trans‐gnetin H is not commercially available and cannot meet the dosage required for the xenograft model. Therefore, we cannot reveal the significance of trans‐gnetin H in vivo. In future studies, we will use mouse models and xenograft tumours (lung and colorectal cancer) to demonstrate the physiological function of trans‐gnetin H—inhibiting mTORC1 activity and promoting autophagy in vivo. As mTORC1 plays a critical role in severe diseases, particularly cancer, our findings are important for understanding the pathogenesis and progress of various diseases.

## 5 CONCLUSION

In summary, we have used a variety of biochemical and cell‐based methods to demonstrate the presence and functionality of trans‐gnetin H in the mTORC1 pathway and autophagy. With regard to the underlying mechanism, the AMPK pathway was found to be the exclusive regulatory mechanism. Importantly, our experiments demonstrated that trans‐gnetin H‐induced AMPK pathway activation may play a role in regulating the mTORC1 pathway autophagy. Further, the trans‐gnetin H‐mediated mTORC1 inactivation mechanism elucidated in this study may be useful for identifying the therapeutic targets for cancer treatment in the future.

## AUTHOR CONTRIBUTIONS

**Chao Xia**: conceptualization, methodology, writing—original draft, investigation. **Guoyan Wang**: conceptualization, methodology, writing—original draft, investigation. **Lei Chen**: investigation, resources, supervision, project administration and funding acquisition. **Huijun Geng**: methodology, investigation. **Junhu Yao**: resources, project administration and funding acquisition. **Zhangzhen Bai**: conceptualization, writing—review and editing, resources, supervision. **Lu Deng**: conceptualization, methodology, writing—original draft, investigation, writing—review and editing, resources, supervision, project administration and funding acquisition.

## CONFLICT OF INTEREST

The authors declare that they have no conflict of interest.

## Supporting information


**Figure S1.** The purity of trans‐gnetin H.

**Figure S2.** Trans‐gnetin H regulates cell proliferation.

**Figure S3.** Trans‐gnetin H regulates cell autophagy via TFEB.

## Data Availability

The data that support the findings of this study are available from the corresponding author upon reasonable request.
