# Supplementary figures and images for: Trans‐gnetin H isolated from the seeds of Paeonia species induces autophagy via inhibiting mTORC1 signalling through AMPK activation
Source: Cell Prolif. 2022 Nov 15;56(3):e13360. doi: 10.1111/cpr.13360 (PMC9977667; doi:10.1111/cpr.13360)

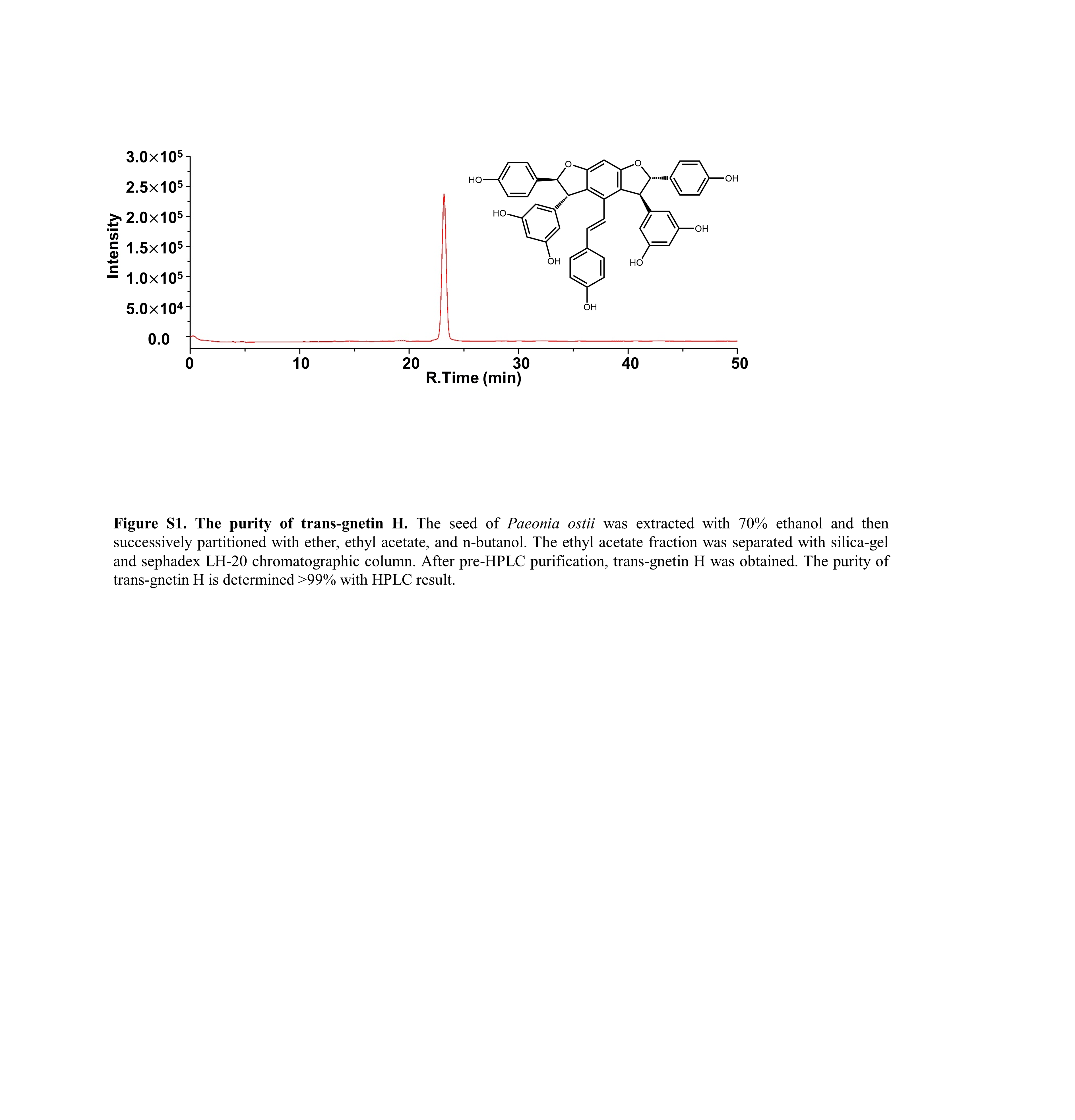

Supplement: Supplementary file 1 — Figure S1. The purity of trans‐gnetin H. [file CPR-56-e13360-s001.TIF]

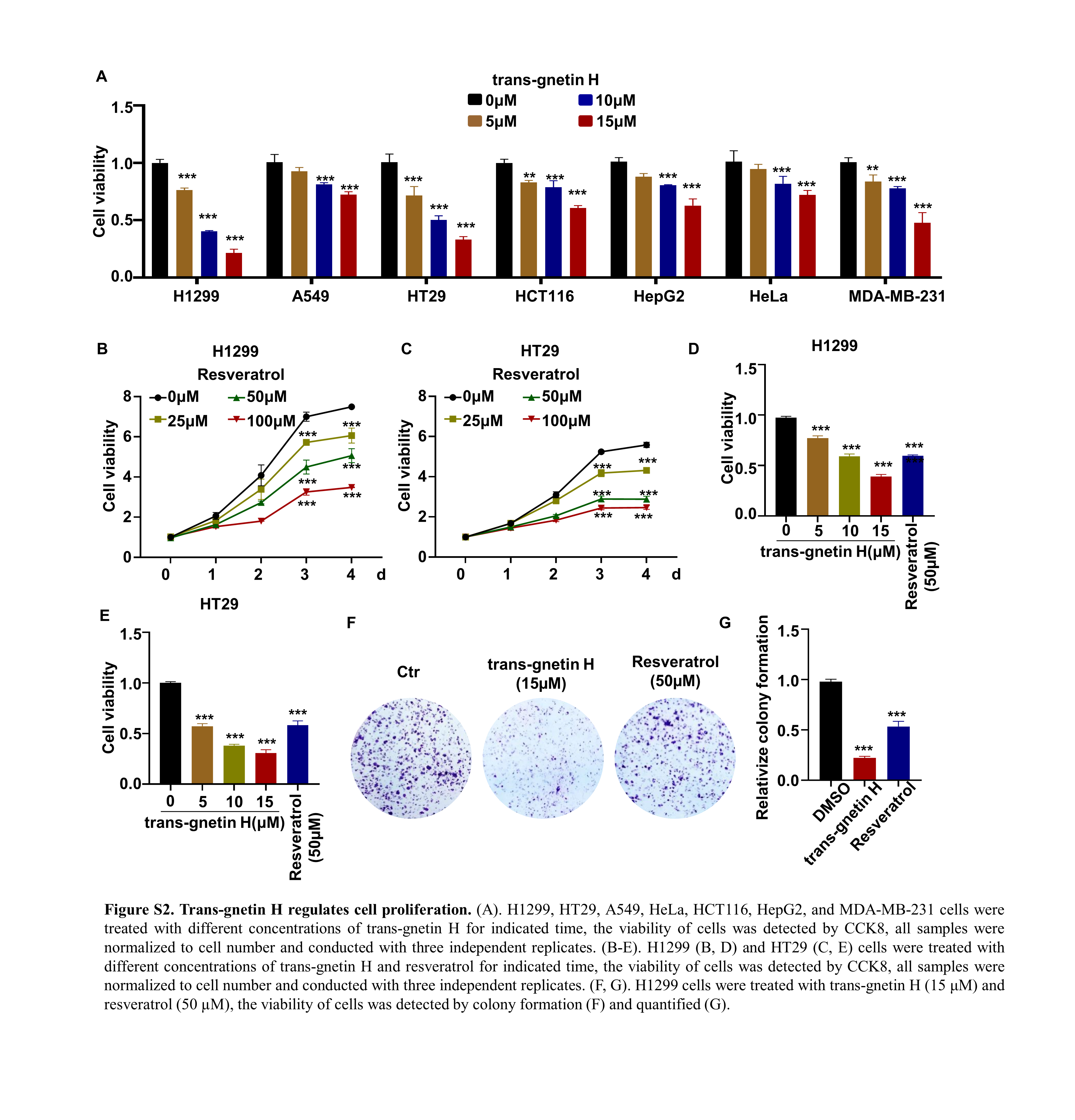

Supplement: Supplementary file 2 — Figure S2. Trans‐gnetin H regulates cell proliferation. [file CPR-56-e13360-s002.TIF]

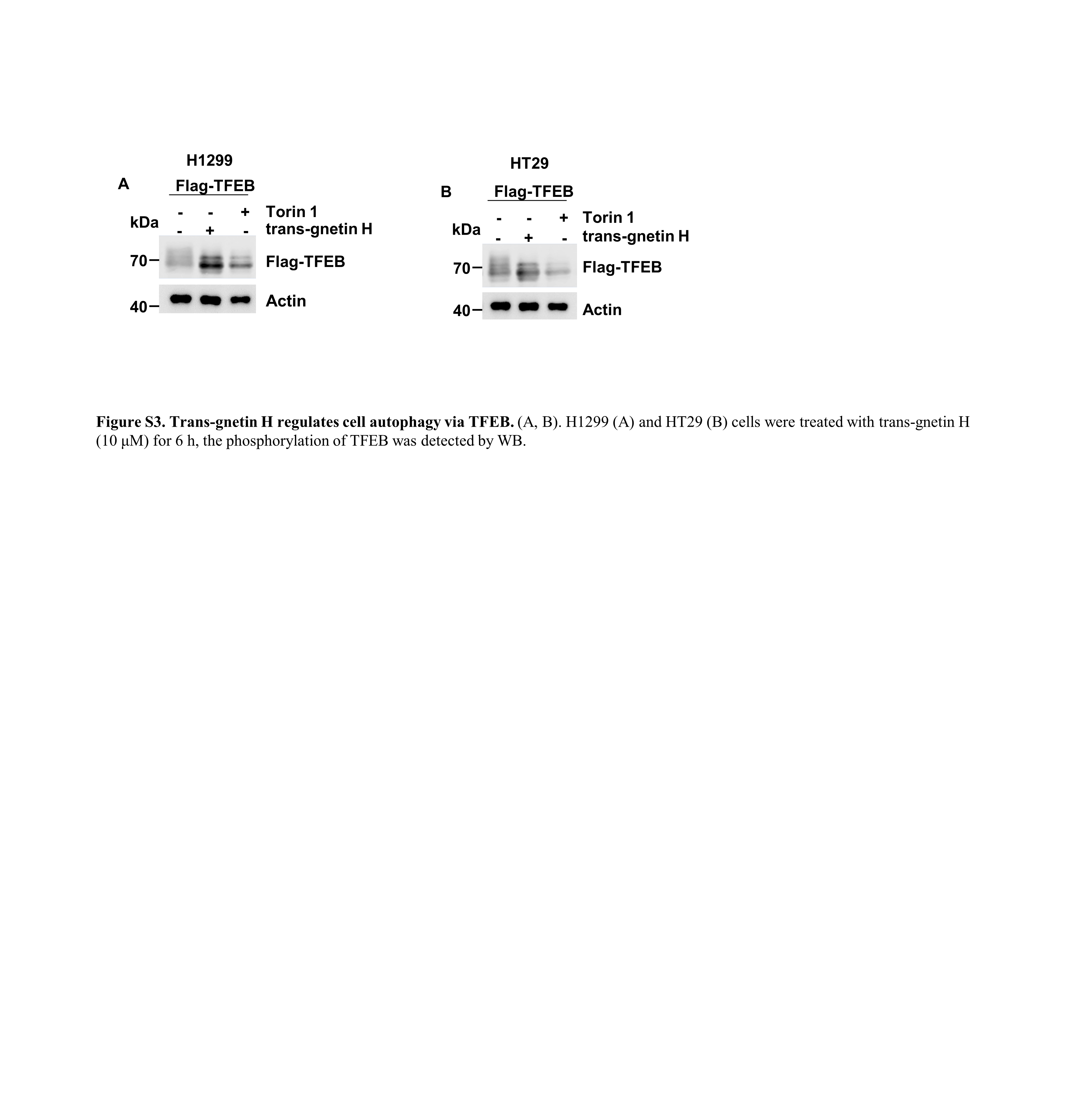

Supplement: Supplementary file 3 — Figure S3. Trans‐gnetin H regulates cell autophagy via TFEB. [file CPR-56-e13360-s003.TIF]
